# Ophthalmic Evaluation and Ocular Candidiasis in Patients with Candidemia: A Retrospective Cohort Study from Thailand

**DOI:** 10.3390/jof12030173

**Published:** 2026-02-27

**Authors:** Sorawit Chittrakarn, Nonthanat Tongsengkee, Siripen Kanchanasuwan, Narongdet Kositpanthawong, Nattapat Sangkakul

**Affiliations:** Division of Infectious Disease, Department of Internal Medicine, Faculty of Medicine, Prince of Songkla University, Songkhla 90110, Thailand; sorawit.c@psu.ac.th (S.C.); tnonthanat@gmail.com (N.T.); kaymed29@yahoo.com (S.K.); narongdet032@gmail.com (N.K.)

**Keywords:** candidemia, ocular candidiasis, candida endopthalmitis, Southeast Asia

## Abstract

**Background:** Ocular candidiasis is a serious metastatic complication of candidemia that may lead to irreversible visual impairment. Although recent meta-analyses suggest an overall prevalence of approximately 10%, real-world data from Southeast Asia remain limited. Regional differences in Candida species distribution, antifungal resistance patterns, and health-care resources may influence both the incidence of ocular candidiasis and the utilization of ophthalmic evaluation in routine practice. **Methods:** We conducted a retrospective cohort study of patients aged ≥15 years with candidemia at a 900-bed tertiary-care university hospital in southern Thailand between January 2014 and August 2025. Only the first episode of candidemia per patient was included. Ophthalmic evaluation was defined as a dilated funduscopic examination performed by an ophthalmologist within 4 weeks of candidemia onset. Ocular candidiasis was classified as Candida chorioretinitis or Candida endophthalmitis according to established definitions. Multivariable logistic regression was used to identify factors independently associated with receipt of ophthalmic evaluation. **Results:** Among 337 patients with candidemia, 67 (19.9%) underwent ophthalmic evaluation. Ocular candidiasis was diagnosed in 9 of 67 evaluated patients (13.4%), corresponding to an overall incidence of 2.7% in the entire cohort. Five patients (7.5%) had Candida chorioretinitis and four (6.0%) had Candida endophthalmitis, including two concordant and two discordant cases. Visual symptoms were assessable in 35 patients, among whom only 4 (11.4%) reported visual complaints; most patients with ocular candidiasis were asymptomatic at diagnosis. *Candida albicans* and *Candida tropicalis* accounted for 55.6% and 44.4% of ocular candidiasis cases, respectively, and bilateral ocular involvement was observed in 77.8%. Ophthalmic findings led to modification of antifungal therapy in 7 of 9 patients with ocular candidiasis (77.8%), most commonly through addition or switching to an azole-based regimen and/or prolongation of treatment duration. In multivariable analysis, vasopressor use at candidemia onset was independently associated with a lower likelihood of ophthalmic evaluation, whereas early infectious diseases consultation was independently associated with increased odds of receiving ophthalmic evaluation. **Conclusions:** In this Southeast Asian cohort, ophthalmic evaluation was infrequently performed but yielded clinically actionable findings and frequently altered antifungal management. The observed incidence of ocular candidiasis among examined patients was higher than that reported in Western countries. Underutilization of an ophthalmic evaluation appears to reflect illness severity and care pathway factors rather than low disease burden, suggesting that the true incidence of ocular candidiasis may be underestimated. Integrating ophthalmic evaluation into standardized candidemia care pathways may improve detection of ocular involvement, particularly in resource-limited settings.

## 1. Introduction

Candidemia is a severe bloodstream infection associated with substantial morbidity and mortality, particularly among critically ill and immunocompromised patients [1]. Beyond systemic infection, hematogenous dissemination of *Candida* species can result in metastatic complications, including ocular candidiasis, which encompasses Candida chorioretinitis and endophthalmitis [2]. Ocular candidiasis is a sight-threatening condition that may lead to irreversible visual loss if not promptly recognized and appropriately treated [3].

The role of routine ophthalmic screening in candidemia remains controversial. The 2016 Infectious Diseases Society of America (IDSA) guidelines recommend dilated funduscopic examination in all patients with candidemia [4], whereas the 2021 American Academy of Ophthalmology advises against routine screening in asymptomatic patients [5]. This discrepancy reflects uncertainty regarding the true incidence, clinical relevance, and management impact of ocular involvement in candidemia, particularly in the contemporary era of echinocandin-based therapy.

Recent meta-analyses indicate that ocular candidiasis develops in approximately 10% of patients with candidemia, including Candida endophthalmitis in 3.1%, with higher reported incidence in Japanese and Korean cohorts compared with Western populations [6]. However, real-world data from Southeast Asia remain scarce, and regional differences in species distribution may influence both the incidence and clinical manifestations of ocular involvement. In addition, local patterns of azole non-susceptibility may affect both the risk of ocular candidiasis and antifungal treatment selection [7,8,9].

In Thailand, *Candida tropicalis* predominates and has shown increasing azole non-susceptibility [7]. Although echinocandins are recommended as first-line therapy for candidemia, their limited vitreous penetration raises concerns regarding both the prevention and treatment of ocular involvement [10]. Meanwhile, the use of liposomal amphotericin B is highly constrained by cost. Furthermore, the real-world diagnostic yield and clinical impact of ophthalmology consultation in resource-limited settings remain poorly defined, particularly in contexts characterized by high ICU case mix, potential verification bias from incomplete ophthalmic evaluation, and heterogeneity in process-of-care factors that may substantially influence outcomes.

In this study, we sought to determine the incidence of ocular candidiasis, evaluate patterns and determinants of ophthalmic evaluation, and assess the clinical impact of ocular findings among patients with candidemia at a tertiary-care hospital in Thailand.

## 2. Method

### 2.1. Study Design and Setting

This retrospective cohort study was conducted at Songklanagarind Hospital, a 900-bed university-affiliated tertiary-care center in southern Thailand. The hospital serves as the main referral center for the southern region of the country.

### 2.2. Study Population

Patients aged ≥15 years with at least one positive blood culture for *Candida* spp. between January 2014 and August 2025 were eligible for inclusion. Only the first episode of candidemia for each patient during the study period was included in the analysis.

### 2.3. Definitions

Ophthalmic evaluation was defined as a dilated funduscopic examination performed by an ophthalmologist within 4 weeks of candidemia onset. This time window was chosen because access to ophthalmology consultation was constrained in routine practice, and in some cases examination was deferred until recovery from neutropenia, in accordance with IDSA guideline recommendations.

Ocular candidiasis was defined as ophthalmologist-diagnosed Candida chorioretinitis and/or endophthalmitis based on characteristic funduscopic findings and classified according to established international definitions [11].

1. Candida endophthalmitis (CE) was categorized as concordant or discordant.
  - Concordant CE was defined as (1) Candida chorioretinitis with extension of surrounding inflammation into the vitreous, or (2) vitreous abscess presenting as intravitreal “fluffballs.”
  - Discordant CE included cases that did not meet concordant criteria but were diagnosed as endophthalmitis based on the ophthalmologist’s overall clinical judgment or when diagnostic criteria were not explicitly documented.
2. Candida chorioretinitis (CCE) was defined as focal, deep, white infiltrative chorioretinal lesions without evidence of direct vitreous involvement.
3. Nonspecific fundus lesions included nerve-fiber-layer infarcts, intraretinal hemorrhages, or white-centered hemorrhages (Roth spots) without chorioretinal infiltration or vitreous involvement.

### 2.4. Microbiological Data

In our hospital, each blood culture set consisted of two aerobic blood culture bottles (BD BACTEC™ Plus Aerobic Medium), with 8–10 mL of blood inoculated into each bottle in accordance with institutional protocols. Blood cultures were incubated for up to 5 days using an automated blood culture system.

Species identification of *Candida* isolates was performed using matrix-assisted laser desorption ionization–time of flight mass spectrometry (MALDI-TOF MS; Bruker Microflex LT, Bruker Daltonics, Bremen, Germany). Identification was considered reliable when the highest MALDI-TOF score was ≥2.0, in accordance with the manufacturer’s recommendations.

### 2.5. Statistical Analysis

Continuous variables were summarized as medians with interquartile ranges (IQRs) and compared using the Wilcoxon rank-sum test. Categorical variables were expressed as frequencies and percentages and compared using the chi-square test or Fisher’s exact test, as appropriate.

Multivariable logistic regression analysis was performed to identify factors independently associated with receipt of ophthalmic evaluation. Variables with *p* values < 0.10 in univariable analyses and those considered clinically relevant a priori were included in the multivariable model. Model discrimination was assessed using the area under the receiver operating characteristic curve (AUC).

All statistical analyses were performed using RStudio (https://posit.co/download/rstudio-desktop/, accessed on 3 February 2026). A two-sided *p*-value < 0.05 was considered statistically significant.

### 2.6. Ethical Considerations

The study was conducted in accordance with the Declaration of Helsinki, and the protocol was approved by the Ethics Committee of REC. 68-500-14-1 on 19 November 2025 with a waiver of informed consent due to the retrospective nature of the study.

## 3. Results

### 3.1. Baseline Characteristics

During the study period, 337 adult patients with candidemia met the inclusion criteria and were included in the analysis. The median age was 60.8 years (IQR, 48.5–72.5), and 174 patients (51.6%) were male.

### 3.2. Receipt of Ophthalmic Evaluation

Among the 337 patients, only 67 (19.9%) underwent ophthalmic evaluation by an ophthalmologist within 4 weeks of candidemia onset.

Baseline characteristics stratified by receipt of ophthalmic evaluation are shown in Table 1. Patients who underwent ophthalmic evaluation were less likely to require vasopressor support at candidemia onset (20.9% vs. 37.8%, *p* = 0.010) and more likely to have diabetes mellitus (20.9% vs. 10.0%, *p* = 0.021). Other demographic characteristics, comorbidities, and severity-of-illness markers, including ICU admission, mechanical ventilation, neutropenia, and national early warning score (NEWS), were similar between groups.

*Candida tropicalis* was the most common species overall (40.9%), followed by *Candida albicans* (30.3%), *Candida glabrata* (14.2%), *Candida parapsilosis* (8.9%) and other Candida species (5.6%). Non-*albicans Candida* species accounted for 69.7% of all candidemia episodes. Polymicrobial bloodstream infection occurred in 32 patients (9.5%) and did not differ significantly between patients who did and did not undergo ophthalmic evaluation.

### 3.3. Care Pathway, Treatment, and Outcomes

Care pathway characteristics, antifungal treatment, and clinical outcomes stratified by receipt of ophthalmic evaluation are summarized in Table 2. Patients who underwent ophthalmic evaluation were more likely to receive early infectious disease (ID) consultation within 48 h (31.3% vs. 19.6%, *p* = 0.048) and within 14 days (44.8% vs. 30.0%, *p* = 0.029).

Early initiation of antifungal therapy was more frequently observed among patients who underwent ophthalmic evaluation, with a higher proportion starting treatment within 72 h (61.2% vs. 44.8%, *p* = 0.020). Amphotericin B deoxycholate was the most commonly prescribed empirical antifungal agent (49.3%), followed by echinocandins (13.6%) and fluconazole (11.9%); notably, 25.0% of patients did not receive antifungal therapy. The distribution of initial antifungal classes was comparable between groups. Among treated patients, amphotericin B constituted 65.9% of antifungal therapy.

Clinical outcomes differed significantly by receipt of ophthalmic evaluation. Patients who underwent ophthalmic evaluation had lower early mortality, including 7-day mortality (4.5% vs. 41.1%, *p* < 0.001), as well as lower in-hospital, 14-day, and 30-day mortality, despite a longer median length of hospital stay (41 vs. 25 days, *p* < 0.001). Rates of persistent candidemia and central venous catheter removal within 7 days did not differ significantly between groups.

### 3.4. Factors Associated with Receipt of Ophthalmic Evaluation

In multivariable logistic regression analysis (Table 3), vasopressor use at candidemia onset was independently associated with a lower likelihood of receiving ophthalmic evaluation. In contrast, early ID consultation was independently associated with increased odds of ophthalmic evaluation. Neutropenia and the presence of CVC were not significantly associated with receipt of ophthalmic evaluation.

### 3.5. Ophthalmic Findings and Management Impact

Among the 67 patients who underwent ophthalmic evaluation, ocular candidiasis was diagnosed in 9 patients (13.4%). Of these, 5 patients (7.5%) were diagnosed with Candida chorioretinitis and 4 patients (6.0%) with Candida endophthalmitis. Among cases of endophthalmitis, two were classified as concordant and two as discordant.

Visual symptoms were assessable in 35 patients (52.2%), among whom only 4 (11.4%) reported visual complaints at the time of examination. Most patients with ocular candidiasis were asymptomatic at diagnosis.

Among the 9 patients diagnosed with ocular candidiasis, *Candida albicans* and *Candida tropicalis* were the predominant causative species. Ocular candidiasis due to *C. albicans* was identified in 5 patients (55.6%), whereas *C. tropicalis* accounted for 4 cases (44.4%). No cases of ocular candidiasis caused by other *Candida* species were observed. Bilateral ocular involvement was present in 7 of 9 patients (77.8%).

Ophthalmic findings led to modification of antifungal therapy in 7 of 9 patients with ocular candidiasis (77.8%), most commonly through addition or switching to an azole-based regimen and/or prolongation of antifungal treatment duration. Intravitreal amphotericin B was administered to two patients. No patients underwent vitrectomy or enucleation (Table 4).

In the remaining two patients, no modification of antifungal therapy was made because of rapid clinical deterioration leading to death or a transition to comfort-focused care before treatment changes could be implemented.

### 3.6. Visual Outcomes Among Patients with Ocular Candidiasis

Individual clinical characteristics, ophthalmic diagnoses, management strategies, and visual outcomes of patients with ocular candidiasis are detailed in Table 5. Among six patients with documented follow-up, three improved to baseline vision, one remained stable, and two experienced worsening visual outcomes. Three patients died before visual outcomes could be assessed.

## 4. Discussion

In this retrospective cohort study, we demonstrated that ophthalmic evaluation was infrequently performed among patients with candidemia in resource-limited settings, yet yielded clinically meaningful findings and frequently influenced antifungal management. Ocular candidiasis was identified in 13.4% of patients who underwent ophthalmic evaluation, and ophthalmic findings led to modification of antifungal therapy in more than three-quarters of affected patients. Importantly, most patients with ocular candidiasis were asymptomatic at the time of diagnosis, highlighting the limitations of symptom-based screening alone.

Our study adds important real-world data from Southeast Asia, a region that has been underrepresented in prior studies of ocular candidiasis. The incidence of ocular candidiasis among examined patients in our cohort (13.4%) is comparable to the pooled global prevalence reported in recent meta-analyses (approximately 10.7%). Notably, the prevalence of concordant Candida endophthalmitis in our study (3.0%) was similar to pooled estimates from Asian cohorts, data that have thus far been derived almost exclusively from Japan and South Korea, and higher than estimates reported from Western countries, where concordant endophthalmitis occurs in approximately 1.4% of candidemia cases.

These findings are particularly noteworthy given the marked differences in Candida species distribution between Thailand and other Asian countries. In our cohort, *Candida tropicalis* was the predominant species, accounting for approximately 40% of candidemia episodes, whereas studies from Japan and South Korea have reported *C. albicans* as the dominant species (40–70%), with *C. tropicalis* comprising only 4.6–17% of isolates [3,12,13,14,15,16]. Although *C. albicans* has been more frequently associated with ocular involvement, potentially due to species-specific virulence factors such as enhanced tissue invasion and stronger host inflammatory activation that promote ocular seeding and damage [17,18], the overall rate of ocular candidiasis in our Thai cohort was comparable to that reported in settings where *C. albicans* predominates. Differences in species distribution may therefore only partially explain geographic variation in reported ocular candidiasis rates, and additional factors, including underlying diseases, host genetic susceptibility and differences in health care delivery and clinical care pathways between developing and high-income settings, may further contribute to these observed disparities [6,19].

Owing to emerging rates of azole resistance in Thailand, particularly among *C. tropicalis*, amphotericin B or echinocandins are recommended as primary agents for the treatment of candidemia; however, the high cost of echinocandins limits their routine use, resulting in frequent reliance on amphotericin B deoxycholate in clinical practice. Accordingly, amphotericin B deoxycholate was used as the initial antifungal therapy in 65% of patients in our cohort, a substantially higher proportion than that reported in other cohorts, in which echinocandins or fluconazole were used as first-line agents in more than 80% of cases [13,14,16,20]. Despite concerns regarding the limited intraocular penetration of amphotericin B, as the drug is not detectable in noninflamed eyes [21,22], we did not observe a higher rate of ocular candidiasis compared with cohorts predominantly treated with fluconazole or echinocandins. This finding is consistent with previous studies suggesting that echinocandins, despite their limited ocular pharmacokinetic properties, are not associated with an increased incidence of ocular candidiasis [13,23]. Moreover, prior prospective data have shown that ocular lesions may develop despite ongoing systemic antifungal therapy, suggesting that antifungal exposure or pharmacokinetics alone may not fully determine the risk of ocular involvement [24]. Taken together, these observations support the concept that ocular candidiasis risk is multifactorial and reinforce the importance of systematic ophthalmic evaluation irrespective of the initial antifungal regimen. Further adequately powered studies are needed to better define the association between initial amphotericin B deoxycholate therapy and the risk of ocular candidiasis.

The clinical value of ophthalmic evaluation extended beyond antifungal selection alone. In our cohort, ophthalmic findings frequently altered the overall clinical trajectory by prompting closer monitoring, reassessment of treatment response, and more structured follow-up, particularly after hospital discharge. Detection of ocular involvement also served as a marker of disseminated infection severity, often influencing multidisciplinary decision-making between infectious diseases specialists and ophthalmologists. In this context, ophthalmic evaluation functioned not merely as a diagnostic tool but as a clinically meaningful intervention that informed ongoing management and risk stratification in patients with candidemia.

The low rate of ophthalmic evaluation observed in our study reflects substantial underutilization in routine clinical practice. At our institution, there is no standardized candidemia care bundle or explicit local guideline mandating ophthalmic evaluation or early infectious diseases consultation. Consequently, ophthalmic evaluation appears to be selectively performed in patients who are clinically stable and able to tolerate examination. This is supported by our finding that patients requiring vasopressor support at candidemia onset were significantly less likely to undergo ophthalmic evaluation, suggesting that critically ill patients are frequently deprioritized.

Patients who did not undergo ophthalmic evaluation experienced substantially higher short-term mortality. This difference likely reflects survivor bias and severity bias, as patients who died early often had no opportunity to receive ophthalmic evaluation, and this group likely included individuals at higher risk for ocular involvement. As a result, the observed incidence of ocular candidiasis in our cohort probably underestimates the true burden of disease. Importantly, early infectious diseases consultation was independently associated with increased odds of ophthalmic evaluation, underscoring the role of multidisciplinary care in improving adherence to recommended candidemia management practices. These findings are consistent with previous studies demonstrating that infectious diseases consultation is associated with a higher likelihood of ophthalmology referral and may lead to increased detection of endophthalmitis [25,26,27].

Our study has several strengths. To our knowledge, this is the first study specifically evaluating ocular candidiasis in Southeast Asia, a region with distinct *Candida* species distribution and emerging azole resistance patterns that differ substantially from those reported in Japan, South Korea, and Western countries. The detailed assessment of care pathways, management impact, and outcomes provides a pragmatic view of real-world practice in a resource-limited setting. However, several limitations should be acknowledged. First, the retrospective design introduces potential verification bias, as not all patients underwent ophthalmic evaluation. Second, the small number of ocular candidiasis cases limited statistical power to identify independent risk factors for ocular involvement. Third, this was a single-center study, which may limit generalizability to other settings. Care pathways and referral practices for ophthalmic evaluation may vary across institutions within Thailand and other Southeast Asian countries. Nevertheless, the predominance of non-*albicans* Candida, particularly *C. tropicalis*, observed in our cohort is consistent with regional epidemiologic patterns, suggesting that the microbiological context of our findings may be broadly applicable to similar settings. Finally, because ophthalmic evaluation was not routinely performed, the true incidence of ocular candidiasis in our population remains uncertain and is likely underestimated.

In conclusion, ophthalmic evaluation in patients with candidemia was markedly underutilized in routine clinical practice but yielded clinically actionable findings with important implications for antifungal management. In resource-limited settings with high burdens of non-*albicans Candida* and restricted antifungal options, ophthalmic evaluation should be considered an integral component of comprehensive candidemia care. Future prospective studies with systematic ophthalmic screening are needed to refine risk stratification and optimize screening strategies.

## Figures and Tables

**Table 1:** Baseline characteristics of patients with candidemia stratified by receipt of ophthalmic evaluation.

Variable	No Ophthalmic Evaluation (n = 270)	Ophthalmic Evaluation (n = 67)	Total (n = 337)	*p* Value
**Demographics**				
Age, years, median (IQR)	61.0 (49.6–72.5)	60.7 (43.4–72.4)	60.8 (48.5–72.5)	0.418
Male sex, n (%)	142 (52.6)	32 (47.8)	174 (51.6)	0.497
**Comorbidities, n (%)**				
Diabetes mellitus	27 (10.0)	14 (20.9)	41 (12.2)	**0.021**
Chronic kidney disease	23 (8.5)	10 (14.9)	33 (9.8)	0.165
COPD	9 (3.3)	2 (3.0)	11 (3.3)	1.000
Chronic heart disease	32 (11.9)	6 (9.0)	38 (11.3)	0.666
Cirrhosis	19 (7.0)	3 (4.5)	22 (6.5)	0.587
Solid malignancy	65 (24.1)	13 (19.4)	78 (23.1)	0.518
Hematologic malignancy	40 (14.8)	10 (14.9)	50 (14.8)	1.000
HIV infection	2 (0.7)	1 (1.5)	3 (0.9)	0.487
Autoimmune disease	7 (2.6)	3 (4.5)	10 (3.0)	0.423
**Risk factors (≤30 days), n (%)**				
Recent hemodialysis	55 (20.4)	9 (13.4)	64 (19.0)	0.226
Recent steroid exposure	149 (55.2)	37 (55.2)	186 (55.2)	1.000
Other immunosuppressive exposure	11 (4.1)	3 (4.5)	14 (4.2)	1.000
Previous Candida colonization	70 (25.9)	16 (23.9)	86 (25.5)	0.876
**Clinical status at candidemia onset, n (%)**				
ICU at onset	124 (45.9)	27 (40.3)	151 (44.8)	0.493
Vasopressor use	102 (37.8)	14 (20.9)	116 (34.4)	**0.010**
Mechanical ventilation	136 (50.4)	32 (47.8)	168 (49.9)	0.682
NEWS score, median (IQR)	4.0 (2.0–7.0)	4.0 (2.0–6.0)	4.0 (2.0–7.0)	0.516
ANC at onset (cells/µL), median (IQR)	9161 (3432–15,340)	8016 (4526–11,740)	8727 (3961–15,041)	0.258
Neutropenia (ANC <500) at onset	46 (17.0)	12 (17.9)	58 (17.2)	0.858
Central venous catheter present	141 (52.2)	36 (53.7)	177 (52.5)	0.782
Duration of CVC before candidemia, days, median (IQR)	7.0 (4.0–15.0)	6.0 (3.0–13.5)	7.0 (3.5–15.0)	0.330
**Baseline microbiology, n (%)**				
Polymicrobial bloodstream infection ^a^	25 (9.3)	7 (10.4)	32 (9.5)	0.814
*Candida tropicalis*	108 (40.0)	30 (44.8)	138 (40.9)	0.490
*Candida albicans*	83 (30.7)	19 (28.4)	102 (30.3)	0.768
*Candida glabrata*	41 (15.2)	7 (10.4)	48 (14.2)	0.435
*Candida parapsilosis*	27 (10.0)	3 (4.5)	30 (8.9)	0.229
Other *Candida* spp.	11 (4.1)	8 (11.9)	19 (5.6)	0.032

^a^ Polymicrobial bloodstream infection was defined as isolation of Candida species with at least one bacterial pathogen from blood cultures obtained during the same episode. Abbreviations: ANC, absolute neutrophil count; COPD, chronic obstructive pulmonary disease; CVC, central venous catheter; HIV, human immunodeficiency virus; ICU, intensive care unit; IQR, interquartile range; NEWS, National Early Warning Score.

**Table 2:** Care pathway, treatment, and outcomes of candidemia stratified by receipt of ophthalmic evaluation.

Variable	No Ophthalmic Evaluation (n = 270)	Ophthalmic Evaluation (n = 67)	Total (n = 337)	*p* Value
**Care Pathway**				
ID consultation ≤ 48 h, n (%)	53 (19.6)	21 (31.3)	74 (22.0)	**0.048**
ID consultation ≤ 14 days, n (%)	81 (30.0)	30 (44.8)	111 (32.9)	**0.029**
Days to ID consultation, median (IQR)	1.19 (0.42–2.93)	1.16 (0.54–2.34)	1.18 (0.45–2.81)	0.990
**Antifungal therapy**				
Antifungal started ≤72 h, n (%)	121 (44.8)	41 (61.2)	162 (48.1)	0.020
Initial antifungal:				
Echinocandin, n (%)	32 (11.9)	14 (20.9)	46 (13.6)	0.072
Fluconazole, n (%)	27 (10.0)	13 (19.4)	40 (11.9)	0.055
Amphotericin B deoxycholate, n (%)	126 (46.7)	40 (59.7)	166 (49.3)	0.075
Time to antifungal therapy, hours, median (IQR)	48.8 (31.5–70.7)	49.0 (28.3–68.4)	48.8 (30.6–69.5)	0.861
**Catheter management**				
CVC removed ≤7 days ^a^, n (%)	101 (37.4)	32 (47.8)	133 (39.5)	0.174
**Outcomes**				
Length of hospital stay, days, median (IQR)	25.0 (15.0–39.0)	41.0 (24.5–64.0)	27.0 (17.0–47.0)	**<0.001**
In-hospital mortality, n (%)	147 (54.4)	25 (37.3)	172 (51.0)	**0.014**
7-day mortality, n (%)	111 (41.1)	3 (4.5)	114 (33.8)	**<0.001**
14-day mortality, n (%)	144 (53.3)	16 (23.9)	160 (47.5)	**<0.001**
30-day mortality, n (%)	160 (59.3)	26 (38.8)	186 (55.2)	**0.004**

^a^ Central venous catheter (CVC) removal within 7 days was assessed among patients with a CVC present at candidemia onset. Abbreviations: CVC, central venous catheter; ID, infectious diseases; IQR, interquartile range.

**Table 3:** Multivariable factors associated with receiving ophthalmic evaluation.

Variable	Adjusted OR	95% CI	*p* Value
Vasopressor use at onset	0.33	0.16–0.68	**0.003**
ICU at onset	1.12	0.57–2.23	0.740
ID consultation ≤ 48 h	1.99	1.08–3.68	**0.028**
Neutropenia at onset	1.06	0.52–2.20	0.866
CVC present	1.61	0.82–3.15	0.166

Abbreviations: CI, confidence interval; CVC, central venous catheter; ICU, intensive care unit; ID, infectious diseases; OR, odds ratio.

**Table 4:** Ophthalmic findings, symptoms, management impact and visual outcome among patients with candidemia who underwent ophthalmic evaluation (n = 67).

Variable	n (%)
**Ophthalmic findings**	
Any ocular candidiasis	9 (13.4)
No ocular involvement	58 (86.6)
**Type of ocular candidiasis**	
Candida chorioretinitis	5 (7.5)
Candida endophthalmitis	4 (6.0)
• Concordant	2 (3.0)
• Discordant	2 (3.0)
**Laterality (among ocular candidiasis, n = 9)**	
Unilateral	2 (22.2)
Bilateral	7 (77.8)
Visual symptoms *	
Symptoms assessable	35/67 (52.2)
Visual symptoms present (among assessable)	4/35 (11.4)
Visual symptoms absent (among assessable)	26/35 (74.3)
Not documented (among assessable)	5/35 (14.3)
**Management impact (among ocular candidiasis, n = 9)**	
Any antifungal therapy modification	7 (77.8)
• Addition of systemic azole	4 (44.4)
• Switch to azole-based regimen	2 (22.2)
• Prolongation of antifungal duration	7 (77.8)
Intravitreal amphotericin B	2 (22.2)
Vitrectomy	0 (0.0)
Enucleation	0 (0.0)
**Visual outcomes (among ocular candidiasis, n = 9)**	
Improved to baseline	3 (33.3)
Stable	1 (11.1)
Worsened	2 (22.2)
Not documented	3 (33.3)

* Visual symptoms were classified as not assessable in patients who were unable to reliably report symptoms due to intubation, sedation, decreased level of consciousness, or aphasia. Abbreviations: OC, ocular candidiasis.

**Table 5:** Ocular candidiasis among patients with candidemia (n = 9).

Case	Age	Sex	Candida Species	Visual Symptoms	Ocular Diagnosis	Laterality	Days to Eye Exam	Systemic Antifungal at Diagnosis	OC-Directed Management	Intravitreal Amphotericin B	Visual Outcome
1	35	F	*C. tropicalis*	Blurred vision	Endophthalmitis—concordant	Bilateral	4	Amphotericin B	Prolong duration	Yes	Stable
2	19	M	*C. tropicalis*	Not assessable	Endophthalmitis—concordant	Bilateral	7	Amphotericin B	Add azole + Prolong duration	No	Worsened
3	58	F	*C. albicans*	Not assessable	Endophthalmitis—discordant	Bilateral	6	Echinocandin	Add azole + Prolong duration	No	Worsened
4	38	M	*C. albicans*	No	Chorioretinitis	Bilateral	3	Echinocandin	Add azole + Prolong duration	Yes	Improved
5	45	F	*C. tropicalis*	Not assessable	Endophthalmitis—discordant	Bilateral	4	Echinocandin	No modification	No	Not documented ^b^
6	67	F	*C. tropicalis*	Not documented	Chorioretinitis	Bilateral	3	Amphotericin B	Switch to azole + prolong duration	No	Improved
7	63	M	*C. albicans*	No	Chorioretinitis	Unilateral (right)	16	Fluconazole	Prolong duration	No	Not documented ^b^
8	47	M	*C. albicans*	Not assessable	Chorioretinitis	Bilateral	7	Amphotericin B	No modification	No	Not documented ^b^
9	58	F	*C. albicans*	Not documented	Chorioretinitis	Unilateral (right)	6	Amphotericin B	Switch to azole + prolong duration	No	Improved

^b^ Not documented indicates that visual outcome could not be assessed due to death before ophthalmologic follow-up. Abbreviations: OC, ocular candidiasis.

## Data Availability

The data supporting the findings of this study are available from the corresponding author upon reasonable request. The data are not publicly available due to privacy and ethical restrictions.
